# Transcatheter mitral valve repair in proportionate and disproportionate functional mitral regurgitation—insights from a small cohort study

**DOI:** 10.1007/s12471-021-01583-6

**Published:** 2021-06-08

**Authors:** J. F. Ooms, M. L. Geleijnse, E. Spitzer, B. Ren, M. P. Van Wiechen, T. W. Hokken, J. Daemen, P. P. T. de Jaegere, N. M. D. A. Van Mieghem

**Affiliations:** 1 Department of Interventional Cardiology, Thoraxcenter, Erasmus University Medical Center, Rotterdam, The Netherlands; 2 Department of Echocardiography, Thoraxcenter, Erasmus University Medical Center, Rotterdam, The Netherlands

**Keywords:** Mitral valve, Functional mitral valve insufficiency, Valve repair

## Abstract

**Background:**

Functional mitral regurgitation (FMR) can be subclassified based on its proportionality relative to left ventricular function and end-diastolic volume. FMR proportionality could help identify responders to transcatheter edge-to-edge mitral valve repair (MitraClip) in terms of residual FMR and/or clinical improvement.

**Methods:**

This single-centre retrospective cohort study evaluated the feasibility of determining FMR proportionality in symptomatic heart failure patients with reduced left ventricular function who were treated with MitraClip for ≥ moderate-to-severe FMR. Baseline proportionate (pFMR) and disproportionate FMR (dFMR) were distinguished. Patient characteristics and MitraClip procedural outcomes were described.

**Results:**

From an overall cohort of 81 eligible FMR patients, 23/81 (28%) had to be excluded due to missing transthoracic echocardiogram parameters, 22/81 were excluded based on FMR severity. The remaining cohort, of 36/81 patients (44%), could be classified into dFMR (*n* = 26) or pFMR (*n* = 10). Conduction disorders were numerically increased in dFMR. All cases requiring > 2 clips were in the dFMR group and absence of FMR reduction occurred more frequently with dFMR.

**Point of view/Conclusion:**

Important limitations in terms of imaging acquisition affect the translation of the FMR proportionality concept to a real-world data set. We did observe different demographic and FMR response patterns in patients with proportionate and disproportionate FMR that warrant further investigation.

**Supplementary Information:**

The online version of this article (10.1007/s12471-021-01583-6) contains supplementary material, which is available to authorized users.

## Introduction

As opposed to degenerative mitral regurgitation, treatment of functional mitral regurgitation (FMR) remains subject to debate [1]. FMR is commonly associated with heart failure and is mainly caused by geometrical or functional abnormalities of structures surrounding the intrinsically normal mitral valve [2]. Currently, FMR therapies—medical or otherwise—are directed at reducing or reversing the remodelling process of the left ventricle [3].

Two randomised controlled trials, COAPT [4] and MITRA-FR [5], investigated percutaneous mitral-valve repair with MitraClip (Abbot, Chicago, IL) in patients with systolic heart failure and > moderate FMR. Conflicting results were reported in terms of mortality and heart failure related hospitalisation [4, 5]. In COAPT, MitraClip treatment improved clinical outcome, while in MITRA-FR, no effect of MitraClip was found [6]. Differences in design and patient characteristics partially explain the discrepancies; in COAPT, patients presented with more severe FMR but less dilated left ventricles compared with patients in MITRA-FR.

Grayburn et al. devised a conceptual framework based on FMR proportionality and disproportionality to the dimensions and function of the left ventricle [7]. The hypothesis suggested that in a subgroup of FMR patients, the location and damage of affected left ventricular segments is unequally distributed. Consequently, segments involved in leaflet coaptation could be disproportionately affected, causing excessive FMR. Interventions targeting resynchronisation and/or mitral clipping might prove beneficial in the setting of disproportionate FMR (dFMR) [8]. The average COAPT FMR was deemed disproportionate to left ventricular dimensions and function as opposed to the average FMR in MITRA-FR that seemed proportionate (pFMR) [7].

This retrospective study aimed to evaluate the feasibility of the FMR proportionality concept in clinical practice as it has only been scarcely evaluated in a real-world MitraClip context [9, 10]. Furthermore, it describes patient demographics, procedural characteristics and immediate FMR reductions after MitraClip for patients with pFMR or dFMR.

## Methods

We retrospectively determined FMR proportionality at baseline in all consecutive heart failure patients with reduced left ventricular ejection fraction (LVEF) who were treated with MitraClip for ≥ moderate-to-severe FMR at the Erasmus University Medical Center. Decision to treat was based on multidisciplinary heart team consensus.

Reasons for excluding patients from analysis were previous mitral valve repair or replacement, heart transplant or untreated significant coronary artery disease requiring revascularisation. Every patient signed informed consent for the clip procedure and use of related data in a dedicated database for study purposes. The study did not fall under the scope of the Medical Research Involving Human Subjects Act per Erasmus Medical Center Institutional Review Board.

The main study objective was to classify baseline FMR as proportionate or disproportionate. Patient and procedure characteristics were described per dFMR and pFMR cohort. Degree of post-procedural FMR reduction (≥ 1 grade) and residual FMR at discharge were recorded.

### Echocardiographic assessment

FMR aetiology and severity were evaluated by transthoracic echocardiogram (TTE) using an integrated approach of multiple qualitative and quantitative parameters including left atrial and ventricular size, jet features, pulmonary artery pressures, effective regurgitant orifice area (using the proximal isovelocity surface area [PISA]), and regurgitant volume (RegVol). RegVol was calculated from the effective regurgitant orifice area or alternatively the volumetric method, if the effective regurgitant orifice area was unavailable and in absence of aortic regurgitation [11, 12]. Reasons for the inability to measure effective regurgitant orifice area and regurgitant volumes were recorded.

### Assessment of functional mitral regurgitation proportionality

**Fig. 1:**
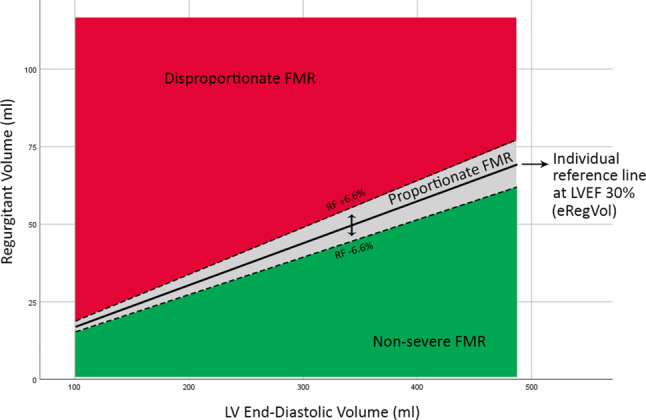
Determining patient-specific proportionality of functional mitral regurgitation. Graph plotting the FMR regurgitant volume vs LVEDV. The black line shows the eRegVol for each given LVEDV at an LVEF of 30% and a regurgitant fraction of 50% (eRegVol = LVEDV × LVEF × 0.50). A margin of ±6.6% was instated (*dotted lines*). Any measured value of regurgitant volume within the *red*, *grey* or *green* zone are deemed disproportionate, proportionate or non-severe respectively. Of note, the reference line shifts for different, individual, values of LVEF. Details on used formulas are found in Appendix I in the Electronic Supplementary Material (ESM). *FMR* functional mitral regurgitation,* LVEDV* left ventricular end-diastolic volume, *LVEF* left ventricular ejection fraction, *eRegVol* expected regurgitant volume

Severe FMR was considered proportionate when the regurgitation was in line with expected RegVol based on left ventricular size and function derived from the Gorlin hydraulic orifice equation [7]. Using left ventricular end-diastolic volume, LVEF and a set regurgitation fraction of 50% we calculated the expected mitral regurgitation volume (eRegVol) of each individual patient. Similarly, we calculated the upper and lower limits of a grey zone around this value using regurgitation fraction ± 6.6% (Fig. 1, Appendix I in the Electronic Supplementary Material [ESM]). [9]. Subsequently, we compared the patient-specific eRegVol with the actual/measured RegVol. A measured RegVol above the eRegVol value was classified as disproportionate, a RegVol within the grey area (approximating the reference line) as proportionate and below as < moderate-to-severe FMR (Fig. 1, Appendix I in the Electronic Supplementary Material [ESM]). Patients with < moderate-to-severe FMR were excluded from the analysis.

### Statistical methods

Continuous variables are presented as mean ± standard deviation for normally distributed data, or as median with interquartile range [IQR] if non-normal. Time-to-event data of one-year mortality and heart failure related hospitalisation is reported using the Kaplan-Meier method. Statistical analyses were performed with SPSS 25.0 (IBM, Armonk, New York).

## Results

**Fig. 2:**
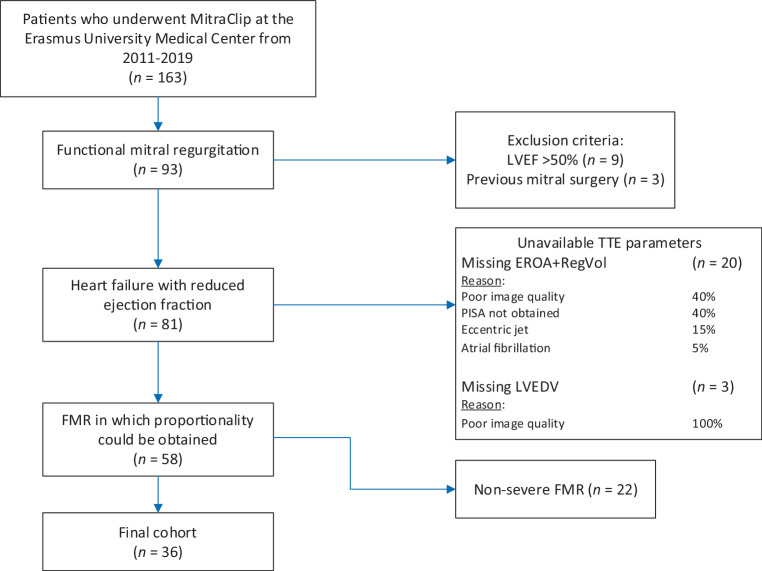
Patient identification. Flow chart of the patient identification process showing that only a small percentage of patients in the prospective Mitraclip database was suitable for determination of proportionality. *EROA* effective regurgitant orifice area, *LVEDV* left ventricular end-diastolic volume, *LVEF* left ventricular ejection fraction, *RegVol* regurgitant volume

**Fig. 3:**
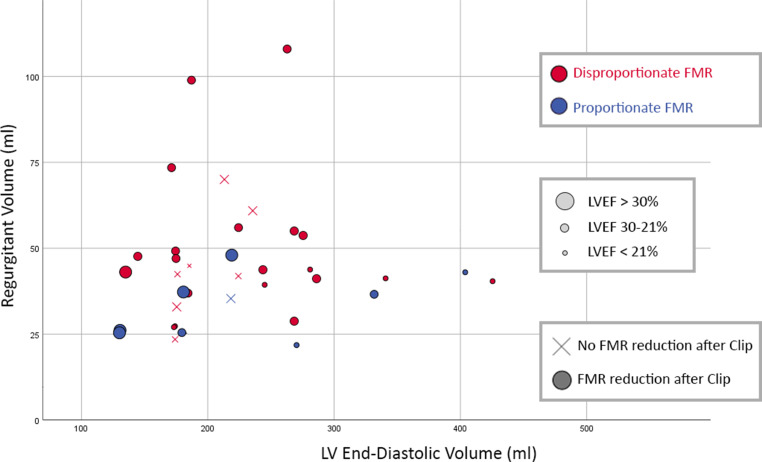
Functional mitral regurgitation severity and proportionality. Scatter plot depicting individual values of FMR regurgitant volume vs LVEDV at baseline. FMR proportionality was determined for 3 different groups of LVEF based on LVEF median and interquartile range (bubble size large, medium and small size depict an LVEF of > 30%, 21–30% and < 21% respectively). Each individual FMR was classified as either disproportionate (*red*) or proportionate (*blue*). Patients with FMR reduction are depicted as a dot and patients without reduction are displayed as a cross. Dot/cross size is coded according to LVEF. *FMR* functional mitral regurgitation,* LVEDV* left ventricular end-diastolic volume, *LVEF* left ventricular ejection fraction

We evaluated all patients who received MitraClip in our centre between 2011 and 2019 (Fig. 2). Ninety-three patients were diagnosed with FMR of which 12 were excluded based on exclusion criteria. FMR proportionality could not be determined in 23/81 patients (28%) because effective regurgitant orifice area, RegVol or left ventricular end-diastolic volume could not be measured based on baseline TTE. In 22/81 patients (27%), FMR was < moderate-to-severe (quantitatively obtained). The final cohort consisted of 36 patients, 26 with dFMR and 10 with pFMR (Fig. 3). Of note, RegVol was derived from the effective regurgitant orifice area in 51 (88%) patients and from the volumetric method in 7 (12%) patients. In the first half of the study period (i.e. ≤ 2015), 16 (44%) patients were included, 20 (56%) patients were included in the second half.

Baseline patient demographics, electrocardiographic and echocardiographic data are reported in Tab. 1 and 2. Procedural results are reported in Tab. 3. Several numerical differences were observed between dFMR and pFMR groups. Older age, kidney dysfunction, higher Society of Thoracic Surgeons (STS) score and prolonged QRS duration was more frequent in dFMR. In dFMR, LVEF and left ventricular end-diastolic volume appeared lower, while total number of clips per procedure and residual FMR severity at discharge were higher (Fig. 3).

**Table 1:** Baseline clinical characteristics

	dFMR (26)	pFMR (10)
*Clinical*		
Age—yr	71.8 ± 10.5	66.2 ± 7.6
Male	17 (65.4)	8 (80.0)
Hypertension	18 (69.2)	7 (70.0)
Diabetes mellitus	8 (30.8)	5 (50.0)
Stroke/TIA	1 (3.8)	–
Peripheral vascular disease	7 (26.9)	2 (20.0)
COPD	3 (11.5)	2 (20.0)
Creatinine clearance—ml/min	38.2 ± 16.3	45.8 ± 11.3
Previous percutaneous coronary intervention	14 (53.8)	6 (60.0)
Previous coronary artery bypass grafting	10 (38.5)	3 (30.0)
Ischaemic cardiomyopathy	20 (76.9)	8 (80.0)
Atrial fibrillation	14 (56.0)	5 (50.0)
Cardiac resynchronisation therapy	7 (26.9)	2 (20.0)
Implantable cardioverter defibrillator	10 (38.5)	5 (50.0)
*NYHA functional class*		
I	–	–
II	3 (11.5)	1 (10.0)
III	14 (53.9)	7 (70.0)
IV	9 (34.6)	2 (20.0)
STS-PROM score, median [IQR]	4 [2–10]	2 [1–5]
*Heart failure medication*		
Renin-angiotensin system antagonist	14 (53.8)	6 (60.0)
Beta-blocker	19 (73.1)	10 (100.0)
Mineralocorticoid antagonist	19 (73.1)	8 (80.0)
Any diuretic	26 (100.0)	10 (100.0)
Any inotropes^a^	–	–

Data is presented in numbers with (%) unless described otherwise. Plus-minus values are means ± SD

*COPD* chronic obstructive pulmonary disease, *dFMR* disproportionate functional mitral regurgitation, *NYHA* New York Heart Association, *pFMR* proportionate functional mitral regurgitation, *TAVI* transcatheter aortic valve replacement, *TIA* transient ischaemic attack, *STS-PROM* Society of Thoracic Surgeons predicted risk of mortality

^a^intravenously administered

**Table 2:** Baseline ventricular conduction and echocardiographic parameters

	dFMR (26)	pFMR (10)
*Baseline ECG variable*		
QRS duration—ms	150 [121–191]	128 [112–186]
QRS duration in non-CRT group—ms	134 [117–182]	123 [111–136]
CRT or QRS ≥ 150 ms	13 (50.0)	3 (30.0)
Left bundle branch block	16 (61.5)	6 (60.0)
*Baseline TTE variable*		
LVEF—%	24.3 ± 1.3	30.2 ± 9.3
LVESV—ml	150 [129–206]	142 [95–227]
LVEDV—ml	200 [175–264]	219 [167–286]
LA volume index—ml/m^2^	76.7 ± 17.7	61.8 ± 14.5
MR grade—no. (%)		
– Moderate-to-severe	7 (26.9)	3 (30.0)
– Severe	19 (73.1)	7 (70.0)
EROA—mm^2^	34.1 [29–43]	22.5 [20–27]
MR VTI—cm	138.9 ± 27.8	148.8 ± 11.0
Mitral RegVol—ml	43 [40–55]	32 [25–39]

Plus-minus values are means ± SD. Other values are median with (interquartile range) or numbers with (%) unless explicitly stated otherwise

*CRT* cardiac resynchronisation therapy, *dFMR* disproportionate functional mitral regurgitation, *ECG* electrocardiogram, *EROA* effective regurgitant orifice area, *LA* left atrium, *LVEDD* left ventricular end-diastolic diameter, *LVEDV* left ventricular end-diastolic volume; *LVEF* left ventricular ejection fraction; *LVESD* left ventricular end-systolic diameter, *LVESV* left ventricular end-systolic volume, *MR* mitral regurgitation,* pFMR* proportionate functional mitral regurgitation, *RegVol* regurgitant volume, *sPAP* systolic pulmonary artery pressure, *TR* tricuspid regurgitation, *TTE* transthoracic echocardiography, *VTI* velocity time integral

**Table 3:** Procedural characteristics

Procedural variable	dFMR (26)	pFMR (10)
*No. clips*		
1	9 (34.6)	5 (50.0)
2	13 (50.0)	5 (50.0)
3	3 (11.5)	–
4	1 (3.8)	–
Technical success^a^	23 (88.5)	10 (100.0)
*Mitral regurgitation at discharge*^b^		
Trace	1 (3.8)	–
Mild	12 (46.1)	4 (40.0)
Mild-to-moderate	2 (7.7)	3 (30.0)
Moderate	5 (19.2)	1 (10.0)
Moderate-to-severe	1 (3.8)	1 (10.0)
Severe	5 (19.2)	1 (10.0)
Discharge MR ≥ moderate^b^	11 (44.0)	3 (33.3)
*Change in MR discharge versus baseline*		
None	5 (19.2)	1 (10.0)
1 Grade	3 (11.5)	2 (20.0)
≥ 2 Grades	18 (69.2)	7 (70.0)
Mitral mean gradient at discharge^b^—mm Hg	3.7 ± 1.7	3.2 ± 1.3

Data is presented in numbers with (%) unless described otherwise. Plus-minus values are means ± SD

*dFMR* disproportionate functional mitral regurgitation, *MR* mitral regurgitation, *pFMR* proportionate functional mitral regurgitation, *MVARC* Mitral Valve Academic Research Consortium criteria

^a^Defined according to MVARC criteria

^b^Obtained with transthoracic echocardiography.

Median follow-up was 464 days (IQR 181–935). One year mortality was 26.5% (*n* = 9). Heart failure related hospitalisation rate within 1 year after MitraClip was 25.7% (*n* = 8). During follow-up, New York Heart Association (NYHA) class improved in 69% of patients (see Tab. 1 in the Electronic Supplementary Material [ESM]).

## Discussion

Our study applied the FMR proportionality concept to heart failure patients who underwent MitraClip therapy in everyday practice and demonstrated that the quality and completeness of echocardiography studies were often suboptimal and lacked essential recordings to determine FMR proportionality. We could categorise only 44% of the overall study cohort into pFMR/dFMR.

Several issues affected the overall sample size of the study. First, methods to determine FMR proportionality rely on effective regurgitant orifice area and RegVol for quantification [9, 10], which may not be straightforward to obtain in FMR [13–15]. In this real-world sample, effective regurgitant orifice area and RegVol appeared missing in a relevant number of cases. Low image quality and absence of essential images required to perform the measurements were the main reasons for missing data. Other methods to derive RegVol were often unavailable or deemed unreliable (i.e. coinciding aortic regurgitation). The minimum amount of image recordings required for proportionality assessment is five (for effective regurgitant orifice area, VTI, left ventricular end-diastolic volume [biplane] and LVEF). Although the requirement of these images and subsequent measurements are established in society guidelines [12, 16], these measurements appeared challenging and often overlooked.

Second, 27% of our initial cohort were excluded because of <moderate-to-severe FMR. This may suggest that the multi-disciplinary heart team may have considered MitraClip as a last resort therapy in the context of end-stage heart failure with < moderate-to-severe FMR. The application of MitraClip for less severe FMR in heart failure is attractive but not the scope of this study.

The ratio between effective regurgitant orifice area and left ventricular end-diastolic volume may be an alternative, simplified method to identify dFMR, yet lacks established cut-off values to separate dFMR from pFMR and disregards LVEF that seemed essential to interpret FMR in the original framework [10]. We, therefore, opted to use patient-specific reference lines derived from each individual’s RegVol, left ventricular end-diastolic volume and LVEF to determine proportionality. Further validation of this FMR proportionality concept requires larger samples and optimised echocardiography protocols to assure complete mitral regurgitation analysis. Clearly, this framework relies on generally adopted, standardised, echocardiographic acquisition protocols including all required images for quantification.

Although applying the proportionality framework to real-world data proved challenging, some interesting observations were made: 1) dFMR was characterised by conduction disorders and more severe left ventricular dysfunction, 2) dFMR may require more clips, and 3) absence of FMR reduction occurred more frequently with dFMR.

Our data seem to confirm that focal left ventricular remodelling and/or dyssynchrony are fundamental mechanisms that underlie dFMR development. There was a higher prevalence of either cardiac resynchronisation therapy implantation or QRS ≥ 150 ms with dFMR. Prolonged QRS duration marks ventricular dyssynchrony [17, 18], while cardiac resynchronisation therapy has been demonstrated to be less efficacious in ischaemic cardiomyopathy [19]. In our dFMR cohort, > 75% had ischaemic heart failure, which might explain persistent cardiac dyssynchrony, non-response to cardiac resynchronisation therapy, and dFMR.

All cases with > 2 clips were in the dFMR group and in spite of using more clips, dFMR reduction appeared less. This finding may result from more extensive mitral regurgitation at baseline. However, clipping does not correct cardiac dyssynchrony, which is associated with residual mitral regurgitation after surgical repair [20].

The retrospective nature of our analysis holds inherent selection bias. To determine FMR proportionality, multiple quantitative echocardiographic parameters are required, each associated with measurement inaccuracies. Importantly, FMR severity in clinical practice is graded using a multi-parametric approach including quantitative and qualitative parameters and is often based on multiple echocardiography studies that may reveal dynamic mitral regurgitation. Standardised comprehensive TTE acquisition protocols and experienced imaging specialists seem essential. This study sample was not powered to make any statistical comparison and should be considered as descriptive and hypothesis generating. We believe our study adds perspective to the application of the concept of mitral regurgitation proportionality in clinical practice and warrants further study. A multicentre initiative is ongoing to complement these single-centre observations.

## Conclusion

Important limitations in terms of imaging acquisition affected the translation of the FMR proportionality concept to a real-world data set. We did observe different demographic and FMR response patterns in patients with proportionate and disproportionate FMR that warrants further investigation.

## Supplementary Information

Appendix I. Determining FMR proportionality

Supplementary Table 1. NYHA functional class during follow-up
