# Supplementary material for: Transcatheter mitral valve repair in proportionate and disproportionate functional mitral regurgitation—insights from a small cohort study
Source: Neth Heart J. 2021 Jun 8;29(7-8):359–64. doi: 10.1007/s12471-021-01583-6 (PMC8271066; doi:10.1007/s12471-021-01583-6)
Supplement: Supplementary file 1 — Appendix I. Determining FMR proportionality [file 12471_2021_1583_MOESM1_ESM.docx]

**Appendix I. Determining FMR proportionality:**

The regurgitant volume (RegVol) was obtained using:

**Regurgitation volume = EROA x MR VTI**

EROA = Effective Regurgitant Orifice Area (calculated with PISA-method)
MR VTI = Mitral Regurgitation Velocity Time Integral

The formula for the expected regurgitant volume (eRegVol) based on left ventricular function and dimensions:

**eRegVol = LVEDV x LVEF x RF**

LVEDV = Left ventricular end diastolic volume
LVEF = Left ventricular ejection fraction
RF = regurgitant fraction (set at 50%*)

*A margin of ±6.6% RF was instated to account for measurement variability and determine proportionality

**RegVol > eRegVol 🡪 Disproportionate FMR**

**RegVol = eRegVol (within grey area) 🡪 Proportionate FMR**

**RegVol < eRegVol 🡪 Non-severe FMR**
