# Supplementary material for: Transcatheter mitral valve repair in proportionate and disproportionate functional mitral regurgitation—insights from a small cohort study
Source: Neth Heart J. 2021 Jun 8;29(7-8):359–64. doi: 10.1007/s12471-021-01583-6 (PMC8271066; doi:10.1007/s12471-021-01583-6)
Supplement: Supplementary file 2 — Supplementary Table 1. NYHA functional class during follow-up [file 12471_2021_1583_MOESM2_ESM.docx]

|  | **dFMR (24)** | **pFMR (8)** |
| --- | --- | --- |
| NYHA functional class – at latest known follow-up |  |  |
| I | 6 (25.0) | 2 (25.0) |
| II | 9 (37.5) | 3 (37.5) |
| III | 5 (20.8) | 2 (25.0) |
| IV | 4 (16.7) | 1 (12.5) |
| Any NYHA functional class improvement during follow-up | 17 (70.8) | 5 (62.5) |

**Supplementary Table 1**

Fractions are of patients with available follow-up. Follow-up was up to 1 year, with latest

known NYHA class reported.

*NYHA* New York Heart Association, *dFMR* disproportionate functional mitral regurgitation,

*pFMR* proportionate functional mitral regurgitation
